# Novel stromal biomarker screening in pancreatic cancer patients using the in vitro cancer-stromal interaction model

**DOI:** 10.1186/s12876-020-01556-w

**Published:** 2020-12-09

**Authors:** Yasunori Nishida, Akiko Kawano Nagatsuma, Motohiro Kojima, Naoto Gotohda, Atsushi Ochiai

**Affiliations:** 1 Division of Pathology, Exploratory Research & Clinical Trial Center, National Cancer Center, Kashiwa, Japan; 2 Department of Hepatobiliary and Pancreatic Surgery, National Cancer Center Hospital East, Kashiwa, Japan; 3 Division of Biomarker Discovery, Exploratory Oncology Research & Clinical Trial Center, National Cancer Center, Kashiwa, Japan; 4 Division of Pathology, Research Center for Innovative Oncology, National Cancer Center Hospital East, 6-5-1 Kashiwanoha, Kashiwa, Chiba 277-8577 Japan

**Keywords:** Pancreatic cancer, Fibroblasts, Tumor microenvironment, Biomarkers

## Abstract

**Background:**

Stromal fibroblasts associated with pancreatic ductal adenocarcinoma (PDAC) play an important role in tumor progression through interactions with cancer cells. Our proposed combination strategies of in vitro and in silico biomarker screening through a cancer-stromal interaction model were previously identified several actin-binding proteins in human colon cancer stroma. The main aim of the present study was to identify novel prognostic markers in human PDAC stroma using our strategies.

**Methods:**

Five primary cultivated fibroblasts from human pancreas were stimulated by two types of pancreatic cancer-cell-conditioned medium (Capan-1 and MIA PaCa-2) followed by gene expression analysis to identify up-regulated genes. Publicly available microarray data set concomitant with overall survival was collected and prognostic marker candidates were selected among the genes that were found to be up-regulated. The mRNA expression levels of the selected genes were evaluated in 5 human fresh PDAC tissues. Finally, survival analysis was performed based on immunohistochemical results on tissue microarrays consisting of 216 surgically resected PDAC tissues.

**Results:**

The microarray data of the cancer-stromal interaction model revealed that 188 probes were significantly regulated in pancreatic fibroblasts. Further, six genes were selected using publicly available microarray data set, and a single Diaphanous-related formin-3 (DIAPH3), actin-binding protein, was identified as a stromal biomarker in PDAC fibroblasts by RNA validation analysis. DIAPH3 exhibited strong immunohistochemical expression in stromal fibroblasts. The high stromal expression of DIAPH3 was associated with shorter survival times of PDAC patients.

**Conclusions:**

DIAPH3 was identified as a prognostic marker in PDAC fibroblasts using our biomarker screening strategies through the cancer-stromal interaction model, indicating that stromal actin-binding proteins might have an important biological role in cancer progression. These strategies were also available in PDAC, and can be used for stromal biomarker screening in various cancers.

## Background

Pancreatic ductal adenocarcinoma (PDAC) is a highly aggressive malignancy with poor clinical prognosis. Despite the recent advances in the cancer treatments, the prognosis of PDAC remains poor. PDAC remains a deadly and by far incurable disease with a 5-year survival rate as low as 9% for all patients and while the survival rates in localized, regional, and distant metastatic diseases were found to be 34, 12, and 3%, respectively [1]. Hence, understanding the molecular complexity of PDAC to aide in biomarker research is required to establish early detection methods and to develop novel therapeutic options for this deadly disease.

One of the most prominent pathological characteristics of PDAC is the development of desmoplastic stroma. Typically, the epithelial component of PDAC constitutes for 20–30% of the tumor volume, whereas remaining tumor volume is made up by stromal cells, extracellular matrix, and soluble signaling mediators [2]. Pancreatic stromal cells included fibroblasts, endothelial cells, immune cells, adipose cells, and nerve cells. Pancreatic fibroblasts were activated through the interaction with cancer cells and played a major role in PDAC progression. Reciprocal interactions between cancer and stromal cells enhanced the metastatic potential and therapeutic resistance to treatments [3–5]. Recently, stromal components, especially fibroblasts in PDAC gathered a lot of attention as potential therapeutic targets [6].

We have reported a novel approach to explore prognostic markers in human cancer stroma using in vitro and in silico biomarker screening strategies based on the cancer-stromal interaction model [7, 8]. Using the cancer-stromal interaction model after cancer-cell-conditioned medium (CCCM) stimulation, we identified up-regulated genes in colonic fibroblasts, which included multiple clinical prognostic markers and were validated using public data and house data available in human colorectal cancer [7, 8]. The up-regulated genes and selected prognostic markers included various actin-binding proteins, which indicated their biological importance in cancer progression. Our main objective in this study was to explore prognostic markers in PDAC stroma using our combination strategies because PDAC is characterized as a prominent stromal component and plays an essential role in tumor progression and metastasis.

## Methods

### General experimental methodology

**Fig. 1:**
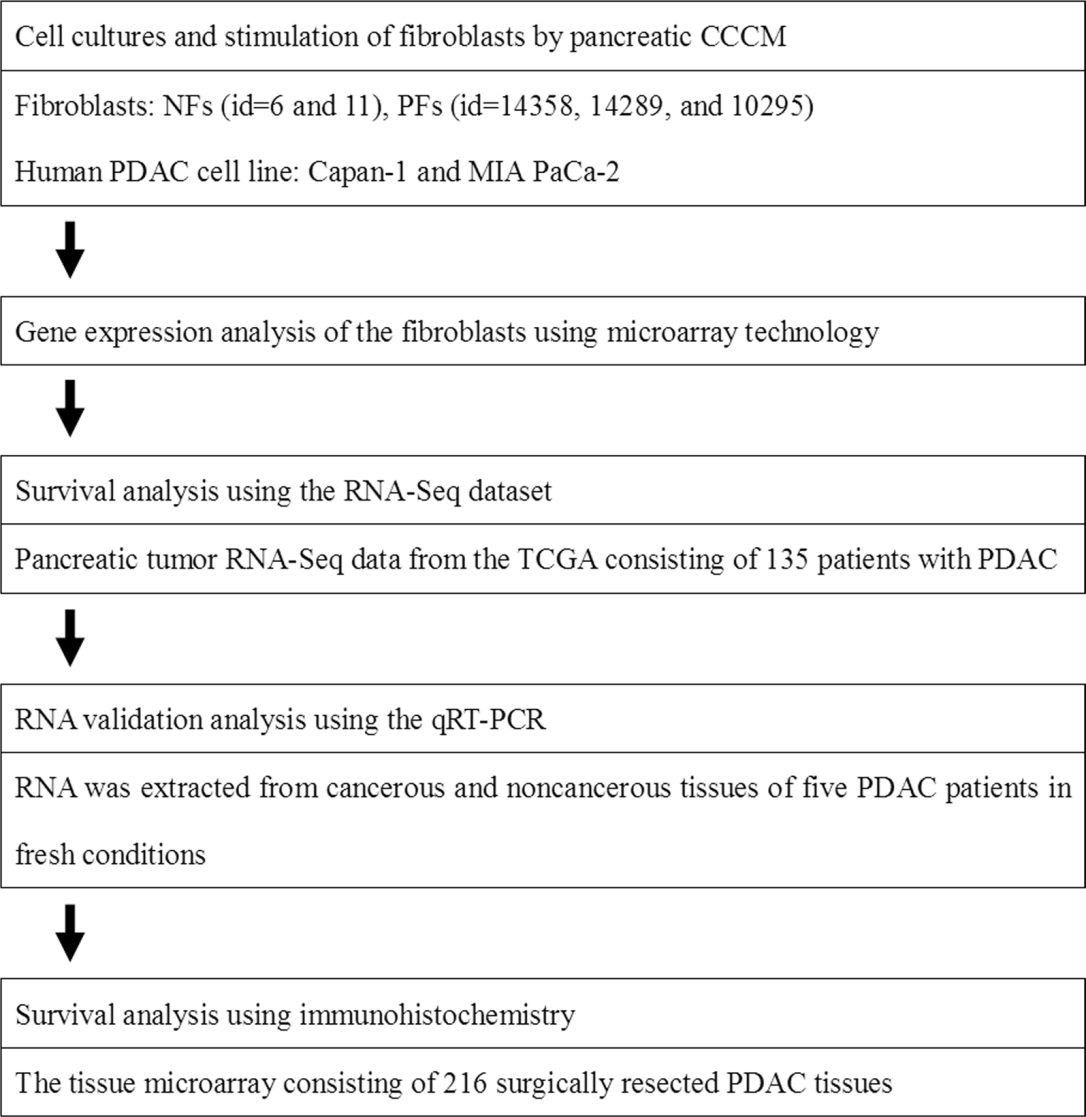
Schematic explaining the study design strategy. Five primary cultivated fibroblasts from human pancreas were prepared and stimulated by two types of pancreatic CCCM (Capan-1 and MIA PaCa-2). Gene expression analysis of the fibroblasts was performed to elucidate up-regulated genes. Gene-expression data concomitant with overall survival in PDAC was collected from the TCGA and prognostic marker candidates were selected from these up-regulated genes. Prognostic marker candidates were confirmed to be expressed in 5 human fresh PDAC tissues compared to normal fresh tissues. Finally, survival analysis using immunohistochemistry on tissue microarrays consisting of 216 surgically resected PDAC tissues was performed. CCCM, cancer-cell-conditioned medium; PDAC, pancreatic ductal adenocarcinoma; TCGA, The Cancer Genome Atlas

Figure 1 illustrated the strategies used in this study. Five primary cultivated fibroblasts from human pancreas were prepared and stimulated by two types of pancreatic CCCM (Capan-1 and MIA PaCa-2) followed by gene expression analysis to identify up-regulated genes. Gene expression data concomitant with overall survival (OS) in PDAC was collected from The Cancer Genome Atlas (TCGA) and prognostic marker candidates were selected among the genes that were found to be up-regulated. Prognostic marker candidates were confirmed to be expressed in 5 human frozen PDAC tissues compared to normal frozen tissues. The last step was to perform survival analysis using immunohistochemistry (IHC) on tissue microarrays (TMAs) consisting of 216 surgically resected PDAC tissues. The study protocol was approved by the Institutional Review Board of the National Cancer Center, Japan (#2016–062).

### Cell lines and cell cultures

Human PDAC cell lines MIA PaCa-2 and Capan-1 were purchased from ATCC (Manassas, VA, USA). Normal fibroblasts (NFs, id = 6 and 11) were obtained from patients undergoing surgery for PDAC as described previously [9]. Three pancreatic fibroblasts (PFs, id = 10295, 14289 and 14358) were purchased from ScienCell Research Laboratories (Carlsbad, CA, USA) (Catalog #3830).

Capan-1 and MIA PaCa-2 were cultured in a Dulbecco’s modified Eagle medium (DMEM) (Sigma-Aldrich, St. Louis, MO, USA) supplemented with 10% fetal bovine serum (FBS) (Gibco, Palo Alto, CA, USA), 100 U/mL penicillin, and 100 μg/mL streptomycin (Sigma-Aldrich, St. Louis, MO, USA) at 37 °C in 5% CO_2_. PFs were maintained in a Stellate Cell Medium (ScienCell Research Laboratories, Carlsbad, CA, USA) at 37 °C in 5% CO^2^, while NFs were maintained in an MF-medium (Toyobo, Tokyo, Japan).

### Stimulation of fibroblasts by pancreatic CCCM

First, 4.5 × 10^4^/cm^2^ of PDAC cells (Capan-1 and MIA PaCa-2) and 9.1 × 10^3^/cm^2^ of fibroblasts (NFs and PFs) were grown separately in DMEM containing 100 U/mL penicillin, 100 μg/mL streptomycin, and 10% FBS for 48 h. Then, PDAC cells and fibroblasts were starved for 24 h. The medium from the starved PDAC cells was obtained as the pancreatic CCCM. At last, the medium in fibroblasts was removed and the pancreatic CCCM or DMEM (without CCCM) was added to stimulate fibroblasts for 24 h. Gene expression levels were comprehensively compared between fibroblasts with and without CCCM stimulation.

### PDAC tissue sample preparation

Total RNA was extracted from the fresh PDAC and non-PDAC tissues of five PDAC patients who underwent surgical resection at the National Cancer Center Hospital East, Kashiwa, Japan. Samples were preserved in RNA*later* (Ambion Inc., Austin, TX) immediately after resection at room temperature and homogenized using a TissueLyser II (QIAGEN, Hilden, Germany) with stainless steel beads for 2 cycles of 3 min at 25 Hz.

### RNA extraction and quality analysis

Total RNA was isolated using an RNeasy Mini Kit (QIAGEN) and RNase-Free DNase Set (QIAGEN) following the manufacturer’s instructions. The RNA quality was assessed using the Agilent RNA 6000 Nano Assay Kits on an Agilent 2100 Bioanalyzer (Agilent Technologies, CA, USA). Samples with > 8.0 FBS values, which indicated clean and intact RNA, were used for microarray gene expression profiling and real-time quantitative PCR (qRT-PCR) assay validation procedures.

### Gene expression analysis using microarray

GeneChip Human Genome U133 Plus 2.0 arrays (Affymetrix, Santa Clara, CA, USA) were used for gene expression analysis. The procedures for target hybridization, washing, and staining for signal amplification were performed following the manufacturer’s instructions. The arrays were scanned with a Gene Chip Scanner 3000 (Affymetrix, Santa Clara, CA, USA). Gene expression data were analyzed with GeneSpring GX12.6 (Agilent Technologies, Santa Clara, CA, USA). Raw data were summarized using microarray suite 5 (MSA5) algorithm and normalized into log-transformed and median centered data to perform the numerical analysis to permit gene selection. *P* values were calculated using one way ANOVA with Benjamini and Hochberg multiple corrections. Genes were selected with the fold change cutoff of > 2.0 and corrected *P* values < 0.05.

### TCGA analysis

To evaluate gene expression in PDAC, publicly available microarray data set of pancreatic tumors were analyzed. This RNA-sequencing (RNA-Seq) dataset was downloaded from the TCGA pancreatic ductal adenocarcinoma dataset on May 2, 2016, from http://cancergenome.nih.gov/. This dataset was built from 183 patients with pancreatic malignancies. Of these, 135 had PDAC without additional histopathological characteristics such as pancreatic neuroendocrine tumors, mucinous colloid carcinomas, and undifferentiated carcinomas. High gene expression was defined as mRNA expression greater than the median expression for each gene. OS was compared between the low expression and high expression groups to explore the clinical implications of the selected gene-specific probes by gene expression analysis of fibroblasts.

### qRT-PCR assay using human pancreas and pancreas cancer tissue

For validation of the selected genes from in vitro and in silico analysis, mRNA and GAPDH levels in PDAC tissues were measured by qRT-PCR using LightCycler® 480 System (Roche Diagnostics, Basel, Switzerland).

The cDNA was synthesized using the PrimScript RT reagent Kit (TaKaRa), and the qRT-PCR was performed in a Light Cycler System (Roche) using SYBR Primix Ex Taq (Tli RNaseH Plus; TaKaRa) according to the manufacturer’s instructions. The target gene expression was normalized with the gene expression of GAPDH.

The mRNA expression levels of selected genes were compared between cancerous and normal tissues of each resected specimen.

### TMA construction

The PDAC TMAs were constructed from surgically resected tissues of 216 PDAC patients at the National Cancer Center Hospital East, Kashiwa, Japan between 2004 and 2014. Patients who underwent preoperative therapy (chemotherapy and/or radiotherapy) were excluded for the purpose of this study. Representative tumor areas were selected and marked on the hematoxylin and eosin (H&E)-stained slides for construction of microarrays. Duplicate cylindrical cores with a diameter of 2.0 mm were prepared from the same tissue block for each case using a manual tissue arrayer (Azumaya Ika Kikai, Tokyo, Japan) and assembled in a tissue microarray format. Serial 4 μm sections were used for immunohistochemical staining.

### IHC staining and evaluation

IHC was performed to evaluate the expression of Diaphanous-related formin-3 gene (DIAPH3) in the TMAs. Immunohistochemical staining was carried out using a fully automated staining instrument; Benchmark ULTRA (Roche Diagnostics, Basel, Switzerland). The rabbit polyclonal DIAPH3 antibody (GTX102892; Gene Tex, Irvine, CA, USA) was used at a dilution of 1:200. For the evaluation of DIAPH3 expression, cytoplasmic staining of PDAC fibroblasts was evaluated. Strong staining of DIAPH3 in stromal fibroblasts was defined as readily visible staining. Comparable IHC staining was observed in lymphocytes, and intensity of stained stromal fibroblasts were judged by reference to those of lymphocytes. Strong staining of DIAPH3 was also defined as the staining in at least 30% of stromal fibroblasts. In order to avoid biased observations, all evaluations of IHC staining in stromal fibroblasts were performed by the same examiner (YN), who was blinded to the surgical outcomes at the time of the evaluation. Representative cases were reviewed by a board-certified pathologist (MK). Patients included in the TMAs were divided into two groups: DIAPH3 weak and strong expression groups.

The clinical characteristics between the two groups were compared using chi-square analysis for non-continuous variables and the Mann-Whitney U test for continuous variables. The Kaplan-Meier method was used to estimate the survival curve, and the log-rank test was used to compare the resulting curves. All the analyses were performed using the IBM SPSS Statistics 21 software package (SPSS Inc., Chicago, IL).

## Results

### Gene expression analysis of fibroblasts

**Fig. 2:**
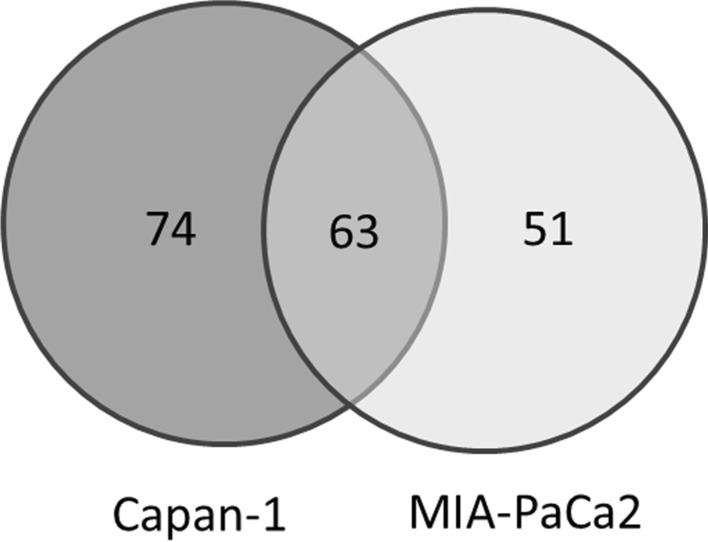
Gene expression analysis of the fibroblasts using microarray technology. Venn diagram demonstrating differentially expressed genes identified from fibroblasts with pancreatic CCCM (Capan-1 and MIA-PaCa2) stimulation. One hundred and eighty-eight probes with the fold change cut-off of > 2.0 and corrected *p*-values < 0.05 in either Capan-1 or MIA-PaCa2 were identified (Additional file 1: Table S1). CCCM, cancer-cell-conditioned medium

Microarray data analysis showed that several genes were upregulated after adding CCCM from Capan-1 and MIA-PaCa2 as shown in Fig. 2. Consequently, 188 probes with the fold change cutoff of > 2.0 and corrected *p*-values < 0.05 in either Capan-1 or MIA-PaCa2 were selected for further analysis (Additional file 1: Table S1).

### Survival analysis using the RNA-Seq data set

Survival analysis using 188 probes from the TCGA RNA-Seq data comprising of 135 PDAC patients was performed. Six genes were selected as candidates for poor prognostic makers, BRIP1 (*p* = 0.031), DIAPH3 (*p* = 0.039), MCM8 (*p* = 0.046), MKI67 (*p* = 0.010), RAD51AP1 (*p* = 0.008), and WDHD1 (*p* = 0.039) (Table 1 and Additional file 2: Fig. S1a, b, c, d, e, f). RAD51AP1 and MKI67 (already known as a prognostic marker [10]) were excluded for further validation analysis.

**Table 1:** Survival analysis using the TCGA RNA-Seq dataset

Gene symbol	Gene ID	*P* value
BRIP1	83990	0.031
DIAPH3	81624	0.039
MCM8	84515	0.046
MKI67	4288	0.010
RAD51AP1	10635	0.008
WDHD1	11169	0.039

*TCGA* The Cancer Genome Atlas, *RNA-seq* RNA sequencing

### Expression of selected genes in human pancreas and pancreas cancer tissue

**Fig. 3:**
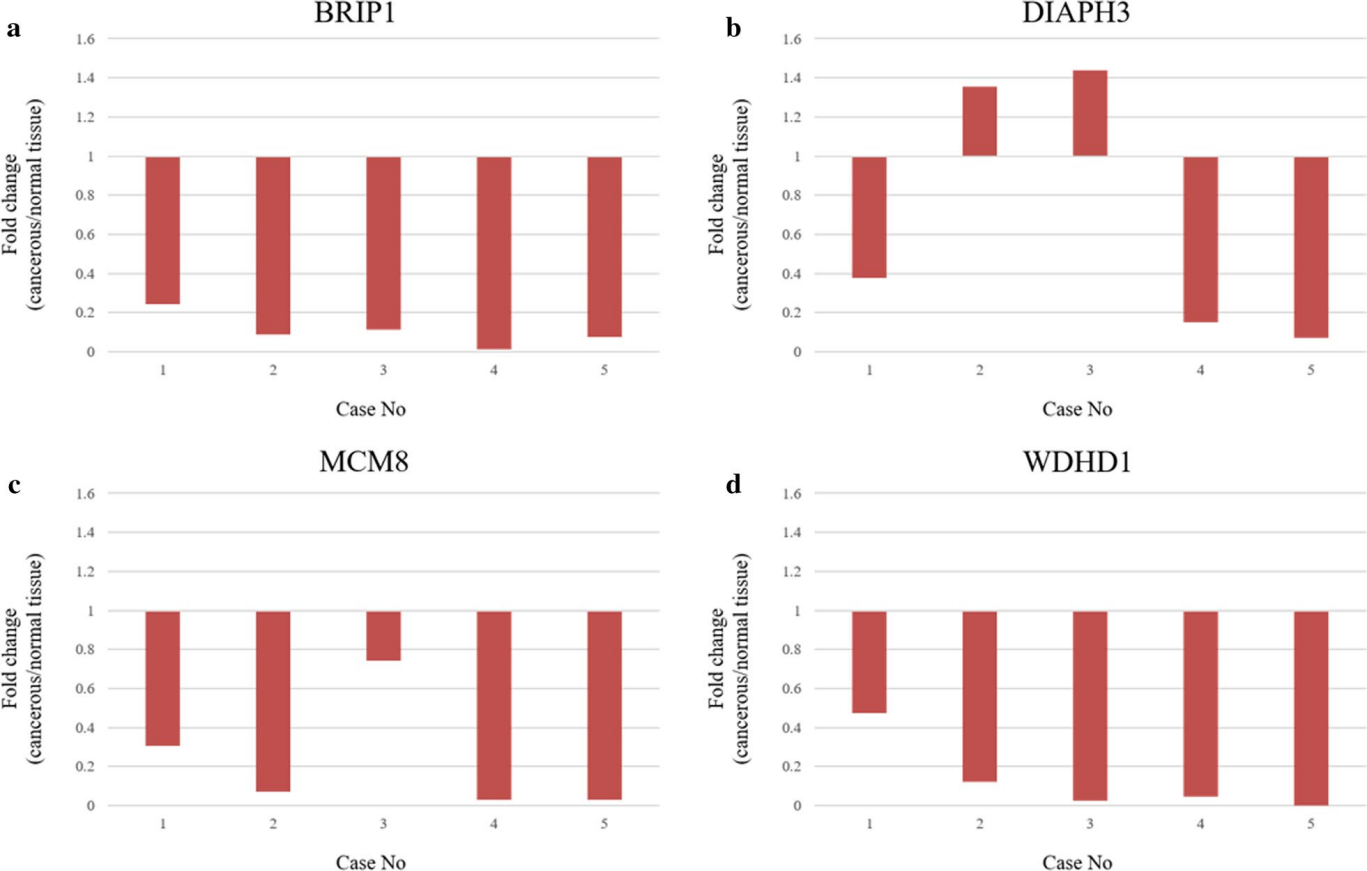
RNA validation analysis using the qRT-PCR. The mRNA expression levels of BRIP1, DIAPH3, MCM8, WDHD1, and GAPDH were evaluated by qRT-PCR in the PDAC and non-PDAC tissues of five resected specimens. Overall fold change was calculated as the median and interquartile-range (IQR): **a** BRIP1 (IQR, 0.07 to 0.11), **b** DIAPH3 (IQR, 0.15 to 1.35), **c** MCM8 (IQR, 0.03 to 0.3), **d** WDHD1 (IQR, 0.02 to 0.12). qRT-PCR, Real-Time Quantitative Reverse Transcription PCR; PDAC, pancreatic ductal adenocarcinoma

The mRNA expression levels of BRIP1, DIAPH3, MCM8, WDHD1, and GAPDH were evaluated by qRT-PCR in the PDAC and non-PDAC tissues of five resected specimens (Fig. 3a, b, c, d). Primer sequences are shown in Additional file 3: Table S2. Upon comparing the mRNA expression levels between cancerous and normal tissues, we found that only DIAPH3 showed different expression levels. DIAPH3 was found to be over-expressed in the cancerous tissue and thus was selected as candidate stromal biomarker in the PDAC tissue (Fig. 3b).

### Survival analysis using immunohistochemistry

**Fig. 4:**
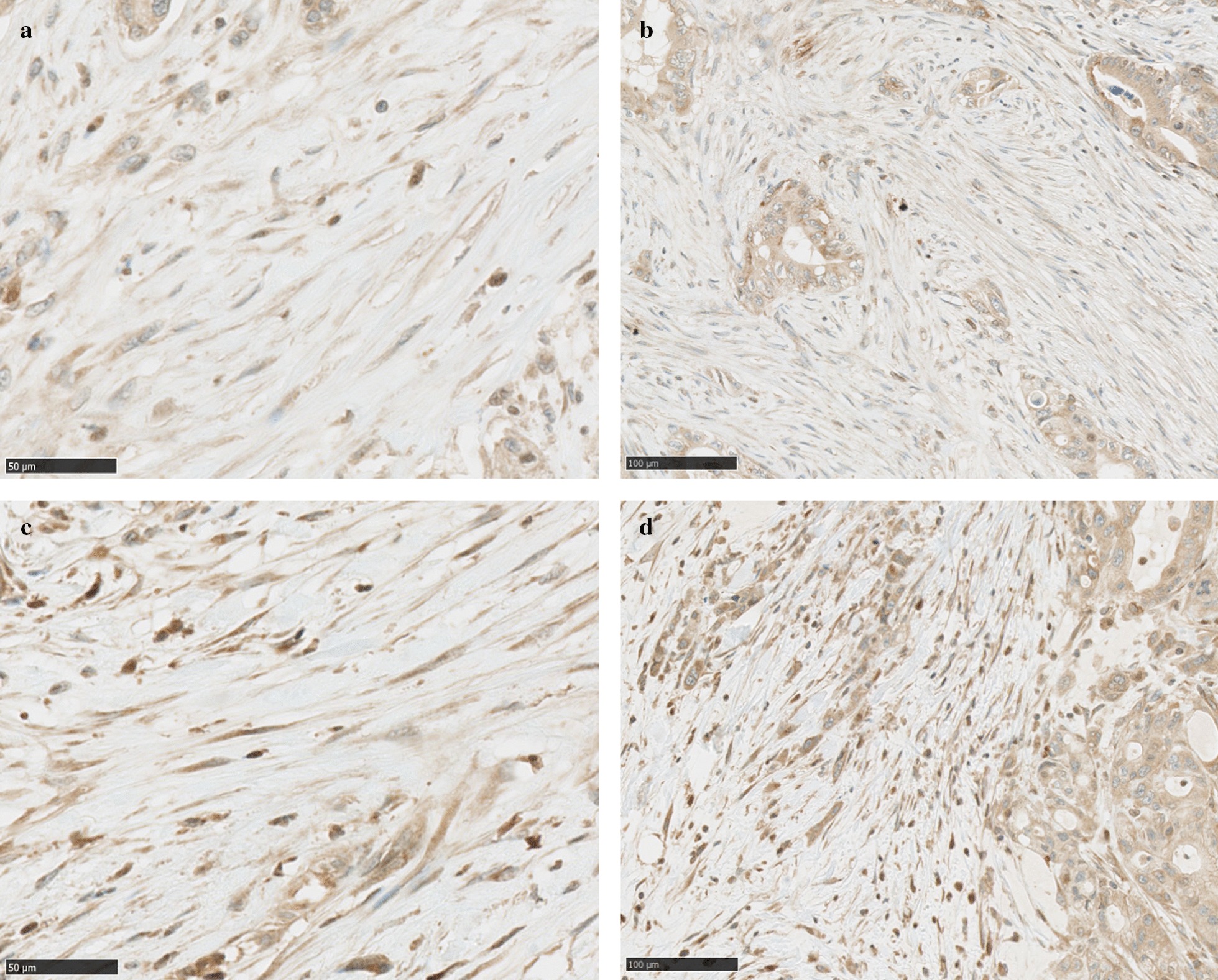
DIAPH3 expression in PDAC fibroblasts. The stromal DIAPH3 expression pattern is depicted in **a**–**b**. Weak (**a**, **b**) and strong (**c**, **d**) expression of DIAPH3 was shown by cytoplasmic staining of PDAC fibroblasts. Strong staining of DIAPH3 in stromal fibroblasts was defined as readily visible staining. Comparable IHC staining was observed in lymphocytes, and intensity of stained stromal fibroblasts were judged by reference to those of lymphocytes. Strong staining of DIAPH3 was also defined as the staining in at least 30% of stromal fibroblasts. DIAPH3, Diaphanous-related formin-3; PDAC, pancreatic ductal adenocarcinoma

IHC analysis revealed that DIAPH3 showed strong expression in the cytoplasm of PDAC fibroblasts (Fig. 4a, b, c, d). 60.6% of stromal fibroblasts exhibited strong cytoplasmic staining for DIAPH3, in addition, comparable IHC staining was observed in lymphocytes and cancer cells (Fig. 4c, d).

Association of clinicopathological parameters and DIAPH3 expression for 216 PDAC patients in our cohort is shown in Additional file 4: Table S3. Patients were divided into two groups based on stromal DIAPH3 expression. We observed that the rates of lymph node metastasis were significantly associated with DIAPH3 expression.

**Fig. 5:**
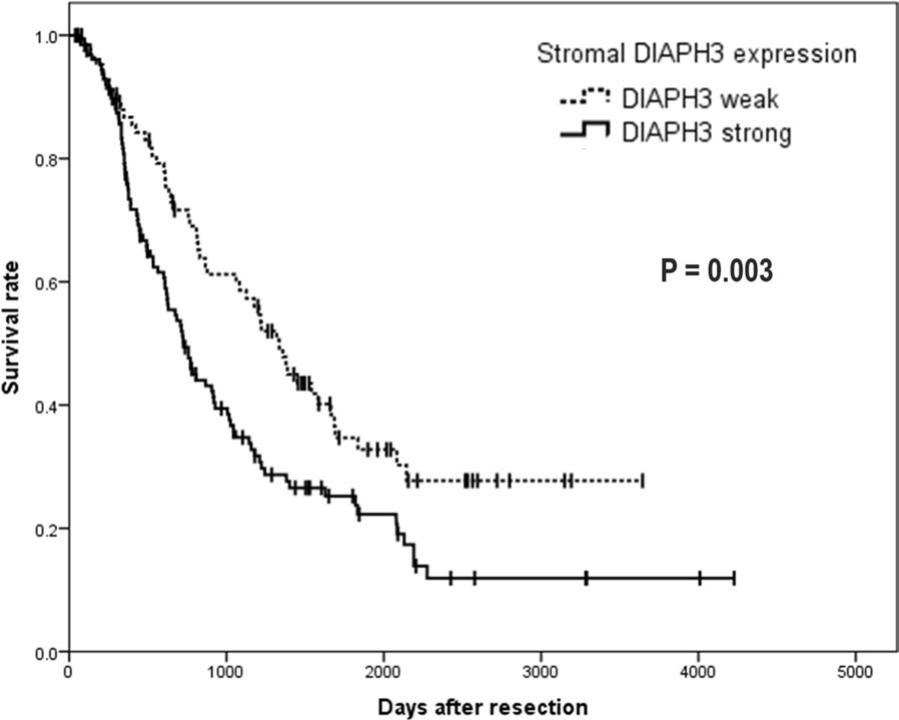
Stromal DIAPH3 expression levels for overall survival analysis. Kaplan-Meier curves showing OS for 216 PDAC patients in TMAs according to stromal DIAPH3 expression levels. Stromal DIAPH3 expression was a negative predictive marker for OS in PDAC patients who underwent surgery. OS, overall survival; PDAC, pancreatic ductal adenocarcinoma; TMA, tissue microarray; DIAPH3, diaphanous-related formin-3

Patients with strong DIAPH3 expression in fibroblasts were associated with poor OS (*p* = 0.003), and we successfully elucidated cancer stromal biomarker through combination framework of in vitro and in silico biomarker screening (Fig. 5).

## Discussion

In this study, we used a combinatorial approach based on in vitro and in silico biomarker screening strategies through the cancer-stromal interaction model [7, 8] for the exploration of biomarkers in PDAC fibroblasts. Subsequent validation studies revealed a new biomarker, DIAPH3 in PDAC fibroblasts. We previously succeeded to identify several stromal biomarkers using similar screening strategies in colorectal cancer [8]. Based on our integrated approach using in vitro and human cancer tissue expression data it will be possible to detect prognostic markers in various cancer stroma. Recently, novel therapies that target the desmoplastic stroma were reported for more effective treatments of PDAC [11, 12]. Identification of stromal biomarkers allows us to select a specific therapeutic target and to better understand mechanisms leading to tumor development. Understanding the biological function of the DIAPH3 in fibroblasts might contribute towards the development of novel therapeutic strategies.

Our previous in vitro and in silico biomarker screening strategies through the cancer-stromal interaction model established that several actin-binding proteins could be used as prognostic markers due to its high expression levels in human colon cancer stroma. Here, we also established that an actin-binding protein DIAPH3 is a novel prognostic biomarker in PDAC fibroblasts. Hager and colleagues defined the role of DIAPH3 as a non-canonical regulator of metastasis that restrained conversion to amoeboid cell behavior in multiple cancer types [13]. DIAPH3 was involved in actin remodeling and regulated cell movement and adhesion. Further, DIAPH3 was reported to be down-regulated in cancer cells and its down-regulation was associated with aggressive or metastatic disease state in various malignancies [13, 14]. However, the presence and significance of DIAPH3 expression in stromal cells have not been assessed. We observed that patients with strong DIAPH3 expression in PDAC fibroblasts had a significantly shorter OS than patients with weak DIAPH3 expression. Interestingly, we previously reported that the expression of the actin-binding protein, transgelin was up-regulated in colorectal cancer stroma and was associated with poor prognosis [8, 15]. On the other hand, transgelin expression in cancer cells have reported to be down-regulated, and was associated with poor prognosis. Therefore, both transgelin and DIAPH3 shared similar biological function of actin-binding to assemble actin cytoskeleton and exhibited opposite clinicopathological relevance between cancer cells and fibroblasts. In this study, the rate of lymph node metastasis was significantly associated with DIAPH3 expression in PDAC fibroblasts. Transgelin was related to lymph node metastasis in colorectal cancer with participation in regulation of the transcriptional program associated with the epithelial-to-mesenchymal transition [16]. Similarly, DIAPH3 might play an important role in tumor metastasis through interactions between stromal fibroblasts and cancer cells.

Generally, actin reorganizations induced by these actin-binding proteins are thought to increase cell stiffness. A recent report using atomic force microscopy revealed that cancer cells had soft properties, which seemed to be consistent with the down-regulation of these actin-binding proteins [17]. On the other hand, cancer tissues are palped as a hard mass [18] and the stiffness is associated with high stromal actin expression. Therefore, we think that our observations regarding the up-regulation of these actin-binding proteins in cancer fibroblasts are directly correlated to practical tumor stiffness and tumor physiology. Based on our results, we speculate that the biological role of actin-binding proteins could be different between cancer cells and fibroblasts.

In this study, we analyzed pancreatic fibroblasts stimulated by tumor cells, and found a common prognostic marker between NFs and PFs. Difference and similarities between NFs and PFs may also reflect heterogeneity and cancer progression of PDAC. We examined the gene expression between NFs and PFs stimulated by tumor cells (Additional file 5: Table S4). Consequently, there seemed to be difference in the expression, and it indicated that the gene expression of the pancreatic fibroblasts was heterogeneous. The heterogeneity of the pancreatic fibroblasts and its influence on cancer evolution should be investigated in the future. In addition, survival data and clinicopathological parameters using IHC has been analyzed retrospectively. This retrospective analysis did not adjust potential confounding factors, including clinical staging, lymph node metastasis, and neural invasion, that might influence the results. Further validation will be needed to establish DIAPH3 as a new prognostic marker for PDAC in a clinical setting.

## Conclusions

We analyzed fibroblasts in PDAC using our biomarker screening strategies through the cancer-stromal interaction model and found a novel prognostic marker in cancer fibroblasts: DIAPH3, actin-binding protein. In conclusion, stromal actin-binding proteins might have an important biological role in cancer progression. We think that in future our biomarker screening strategies will significantly contribute to identifying prognostic markers in various cancer microenvironments. Recently, normalization of tumor microenvironment can be available for the enhancement of efficacy of cytotoxic and immune-oncologic agents [19, 20]. Biomarkers which associated both with fibroblast activation and patient prognosis can be a target for microenvironmental normalization in the future.

## Supplementary Information

**Additional file 1: Table 1**. Selected probe sets by gene expression analysis of fibroblasts with and without pancreatic CCCM stimulation

**Additional file 2: Figure 1**. Kaplan-Meier and log-rank analysis using the TCGA RNA-Seq dataset. After survival analysis of 188 probes using the TCGA data, six genes were selected as prognostic makers: (a) BRIP1 (*p* = 0.031), (b) DIAPH3 (*p* = 0.039), (c) MCM8 (*p* = 0.046), (d) MKI67 (*p* = 0.01), (e) RAD51AP1 (*p* = 0.008) and (f) WDHD1 (p = 0.039). TCGA, The Cancer Genome Atlas; RNA-seq, RNA sequencing.

**Additional file 3: Table 2**. The following primers were used for the qRT-PCR method

**Additional file 4: Table 3**. Association of clinicopathological parameters and stromal DIAPH3 expression of 216 PDAC patients in our cohort

**Additional file 5: Table 4**. Selected probe sets by gene expression analysis of fibroblasts (NFs and PFs) with pancreatic CCCM stimulation

## Data Availability

The datasets used and/or analyzed during the study are available from the corresponding author on reasonable request.

## Abbreviations

PDAC: Pancreatic ductal adenocarcinoma
CCCM: Cancer-cell-conditioned medium
OS: Overall survival
TCGA: The Cancer Genome Atlas
IHC: Immunohistochemistry
TMA: Tissue microarray
NFs: Normal fibroblasts
PFs: Pancreatic fibroblasts
DMEM: Dulbecco’s modified Eagle medium
FBS: Fetal bovine serum
qRT-PCR: Real-time quantitative PCR
RNA-Seq: RNA-sequencing
DIAPH3: Diaphanous-related formin-3 gene
