# Supplementary material for: Novel stromal biomarker screening in pancreatic cancer patients using the in vitro cancer-stromal interaction model
Source: BMC Gastroenterol. 2020 Dec 9;20:411. doi: 10.1186/s12876-020-01556-w (PMC7724826; doi:10.1186/s12876-020-01556-w)
Supplement: Supplementary file 1 — Additional file 1: Table 1. Selected probe sets by gene expression analysis of fibroblasts with and without pancreatic CCCM stimulation [file 12876_2020_1556_MOESM1_ESM.docx]

Supplementary Table 1

Selected probe sets by gene expression analysis of fibroblasts with and without pancreatic CCCM stimulation

| Probe Set ID | Gene Symbol | P value  (Corr) | P value | Fold change  (Capan-1 vs control) | Fold change  (MIA-PaCa2 vs control) |
| --- | --- | --- | --- | --- | --- |
| 1559713_at | AC141928.1 | 0.016 | 0.011 | 1.492 | 2.007 |
| 217211_at | ACTBP9///ACTBP9 | 0.003 | <0.001 | 2.448 | 1.451 |
| 237411_at | ADAMTS6 | 0.001 | <0.001 | 2.528 | 1.753 |
| 220390_at | AGBL2 | 0.007 | 0.003 | 2.761 | 1.415 |
| 206561_s_at | AKR1B10 | 0.001 | <0.001 | 6.720 | 2.748 |
| 213004_at | ANGPTL2 | 0.008 | 0.004 | 2.751 | 1.022 |
| 219514_at | ANGPTL2 | 0.005 | 0.002 | 2.863 | 1.159 |
| 1552619_a_at | ANLN | 0.005 | 0.002 | 3.112 | 6.275 |
| 222608_s_at | ANLN | 0.022 | 0.016 | 2.445 | 6.529 |
| 219636_s_at | ARMC9 | 0.004 | 0.001 | 2.008 | 1.857 |
| 219637_at | ARMC9 | 0.003 | 0.001 | 2.124 | 1.829 |
| 219103_at | ASAP3 | 0.006 | 0.003 | 2.064 | 1.499 |
| 208079_s_at | AURKA | 0.009 | 0.005 | 1.968 | 4.147 |
| 225606_at | BCL2L11 | <0.001 | <0.001 | 2.251 | 1.743 |
| 210334_x_at | BIRC5 | 0.006 | 0.003 | 1.914 | 4.191 |
| 204531_s_at | BRCA1 | 0.014 | 0.009 | 1.986 | 3.735 |
| 214727_at | BRCA2 | 0.007 | 0.003 | 1.688 | 2.958 |
| 221703_at | BRIP1 | 0.017 | 0.012 | 2.504 | 3.623 |
| 235609_at | BRIP1 | 0.014 | 0.009 | 2.771 | 4.069 |
| 215509_s_at | BUB1 | 0.006 | 0.003 | 1.743 | 4.158 |
| 230281_at | C16orf46 | 0.001 | <0.001 | 2.110 | 1.842 |
| 220840_s_at | C1orf112 | 0.019 | 0.014 | 1.285 | 2.036 |
| 229886_at | C5orf34 | 0.013 | 0.008 | 1.837 | 2.279 |
| 209970_x_at | CASP1 | 0.006 | 0.003 | 2.095 | 1.052 |
| 211367_s_at | CASP1 | 0.005 | 0.002 | 2.683 | 1.083 |
| 211368_s_at | CASP1 | 0.006 | 0.003 | 2.401 | 1.039 |
| 203418_at | CCNA2 | 0.019 | 0.014 | 2.014 | 3.493 |
| 205034_at | CCNE2 | 0.019 | 0.013 | 2.519 | 3.541 |
| 211559_s_at | CCNG2 | 0.020 | 0.014 | 2.153 | 1.600 |
| 244352_at | CD84 | 0.033 | 0.028 | 3.596 | 2.062 |
| 203968_s_at | CDC6 | 0.007 | 0.003 | 2.223 | 2.760 |
| 224753_at | CDCA5 | 0.024 | 0.018 | 1.678 | 3.472 |
| 220227_at | CDH4 | <0.001 | <0.001 | 2.692 | 1.848 |
| 203213_at | CDK1 | 0.014 | 0.010 | 2.304 | 8.492 |
| 203214_x_at | CDK1 | 0.010 | 0.005 | 2.037 | 6.069 |
| 204252_at | CDK2 | 0.006 | 0.003 | 1.742 | 2.168 |
| 227775_at | CELF6 | 0.002 | <0.001 | 2.003 | 1.594 |
| 222118_at | CENPN | 0.003 | <0.001 | 2.197 | 2.280 |
| 228559_at | CENPN | 0.003 | 0.001 | 2.059 | 1.865 |
| 218883_s_at | CENPU | 0.008 | 0.004 | 2.138 | 5.902 |
| 229305_at | CENPU | 0.032 | 0.026 | 1.890 | 3.477 |
| 232635_at | CEP128 | 0.003 | 0.001 | 1.935 | 3.969 |
| 244033_at | CEP128 | 0.002 | <0.001 | 2.296 | 4.513 |
| 212675_s_at | CEP68 | <0.001 | <0.001 | 2.104 | 1.532 |
| 205387_s_at | CGB8///CGB7///CGB5///CGB3///CGB1 | 0.004 | 0.001 | 6.203 | 1.443 |
| 235117_at | CHAC2 | <0.001 | <0.001 | 2.070 | 2.167 |
| 229610_at | CKAP2L | 0.001 | <0.001 | 2.948 | 7.253 |
| 235099_at | CMTM8 | 0.004 | 0.001 | 2.409 | 1.434 |
| 242916_at | CNTRL | 0.003 | <0.001 | 3.760 | 1.194 |
| 238732_at | COL24A1 | 0.005 | 0.002 | 4.089 | 1.873 |
| 218168_s_at | COQ8A | 0.001 | <0.001 | 2.389 | 1.642 |
| 225956_at | CREBRF | 0.007 | 0.003 | 2.371 | 1.861 |
| 210764_s_at | CYR61 | 0.006 | 0.002 | 2.015 | 1.400 |
| 228281_at | DDIAS | 0.009 | 0.005 | 2.272 | 4.521 |
| 232596_at | DIAPH3 | 0.041 | 0.035 | 3.504 | 2.871 |
| 218585_s_at | DTL | 0.004 | 0.002 | 3.929 | 9.651 |
| 222680_s_at | DTL | 0.003 | <0.001 | 2.667 | 5.868 |
| 201041_s_at | DUSP1 | 0.004 | 0.002 | 2.121 | 1.444 |
| 233850_s_at | EBF4 | 0.025 | 0.019 | 1.597 | 2.410 |
| 235178_x_at | ESCO2 | 0.008 | 0.004 | 9.307 | 17.988 |
| 38158_at | ESPL1 | 0.048 | 0.042 | 1.535 | 2.596 |
| 204603_at | EXO1 | 0.049 | 0.043 | 1.650 | 3.531 |
| 226431_at | FAM117B | 0.001 | <0.001 | 2.004 | 1.769 |
| 242560_at | FANCD2 | 0.011 | 0.006 | 2.188 | 4.369 |
| 213007_at | FANCI | 0.012 | 0.007 | 1.871 | 3.935 |
| 213008_at | FANCI | 0.015 | 0.010 | 1.423 | 4.091 |
| 48031_r_at | FAXDC2 | 0.002 | <0.001 | 2.836 | 1.741 |
| 232851_at | FBXO3 | 0.030 | 0.024 | 2.142 | 3.732 |
| 204767_s_at | FEN1 | 0.016 | 0.011 | 1.930 | 2.634 |
| 204768_s_at | FEN1 | 0.003 | 0.001 | 2.017 | 2.784 |
| 1555136_at | FGD6 | 0.003 | <0.001 | 4.538 | 5.201 |
| 203592_s_at | FSTL3 | 0.003 | <0.001 | 2.516 | 1.136 |
| 1560129_at | FW340027 | 0.017 | 0.012 | 2.299 | 3.143 |
| 231577_s_at | GBP1 | 0.013 | 0.008 | 2.149 | 1.614 |
| 219920_s_at | GMPPB///AMIGO3 | 0.002 | <0.001 | 2.156 | 1.730 |
| 235387_at | GSTCD | 0.003 | 0.001 | 1.672 | 2.217 |
| 215942_s_at | GTSE1 | 0.006 | 0.003 | 1.834 | 3.897 |
| 206638_at | HTR2B | 0.001 | <0.001 | 6.675 | 2.383 |
| 207826_s_at | ID3 | 0.011 | 0.007 | 2.296 | 1.294 |
| 206926_s_at | IL11 | 0.010 | 0.005 | 2.971 | 1.753 |
| 221985_at | KLHL24 | 0.010 | 0.005 | 2.039 | 1.794 |
| 226158_at | KLHL24 | 0.001 | <0.001 | 2.246 | 1.895 |
| 220324_at | LINC00472 | 0.013 | 0.008 | 3.820 | 3.645 |
| 1554768_a_at | MAD2L1 | 0.006 | 0.003 | 1.919 | 4.186 |
| 221760_at | MAN1A1 | 0.003 | <0.001 | 2.044 | 1.177 |
| 230112_at | MARCH4 | 0.006 | 0.003 | 2.652 | 1.470 |
| 212141_at | MCM4 | 0.005 | 0.002 | 1.487 | 2.014 |
| 212142_at | MCM4 | 0.006 | 0.003 | 1.350 | 2.192 |
| 224320_s_at | MCM8 | 0.001 | <0.001 | 1.517 | 2.286 |
| 204825_at | MELK | 0.006 | 0.003 | 1.893 | 3.599 |
| 207761_s_at | METTL7A | 0.001 | <0.001 | 5.583 | 2.222 |
| 221779_at | MICALL1 | 0.002 | <0.001 | 2.039 | 1.305 |
| 239130_at | MIR101-1///MIR3671 | 0.004 | 0.001 | 1.750 | 2.148 |
| 212020_s_at | MKI67 | 0.011 | 0.006 | 1.638 | 2.850 |
| 212093_s_at | MTUS1 | <0.001 | <0.001 | 3.597 | 1.196 |
| 203359_s_at | MYCBP///GJA9-MYCBP | <0.001 | <0.001 | 2.222 | 2.093 |
| 229339_at | MYOCD | 0.003 | <0.001 | 3.320 | 1.172 |
| 237206_at | MYOCD | 0.011 | 0.006 | 2.864 | 1.549 |
| 213375_s_at | N4BP2L1 | <0.001 | <0.001 | 2.720 | 2.257 |
| 217197_x_at | N4BP2L1 | <0.001 | <0.001 | 2.375 | 2.698 |
| 218662_s_at | NCAPG | 0.015 | 0.010 | 2.000 | 6.072 |
| 219588_s_at | NCAPG2 | 0.038 | 0.032 | 1.516 | 2.936 |
| 211091_s_at | NF2 | 0.001 | <0.001 | 1.817 | 2.013 |
| 218915_at | NF2 | <0.001 | <0.001 | 2.105 | 1.587 |
| 210113_s_at | NLRP1 | 0.002 | <0.001 | 2.158 | 1.298 |
| 206645_s_at | NR0B1 | 0.005 | 0.002 | 3.441 | 1.995 |
| 213599_at | OIP5 | 0.007 | 0.003 | 2.028 | 6.413 |
| 1558906_a_at | OSER1-AS1 | 0.005 | 0.002 | 2.026 | 1.670 |
| 227928_at | PARPBP | 0.045 | 0.039 | 1.456 | 2.270 |
| 225977_at | PCDH18 | 0.002 | <0.001 | 2.945 | 1.811 |
| 202503_s_at | PCLAF | 0.006 | 0.003 | 2.205 | 6.032 |
| 211713_x_at | PCLAF | 0.009 | 0.005 | 2.114 | 4.798 |
| 224399_at | PDCD1LG2 | 0.002 | <0.001 | 3.338 | 2.628 |
| 219304_s_at | PDGFD | 0.001 | <0.001 | 5.201 | 2.722 |
| 222860_s_at | PDGFD | 0.003 | <0.001 | 3.777 | 2.589 |
| 200737_at | PGK1 | 0.005 | 0.002 | 1.941 | 2.253 |
| 227068_at | PGK1 | 0.001 | <0.001 | 1.975 | 2.132 |
| 221756_at | PIK3IP1 | 0.003 | <0.001 | 2.734 | 1.932 |
| 221757_at | PIK3IP1 | 0.004 | 0.002 | 2.182 | 1.854 |
| 205479_s_at | PLAU | 0.006 | 0.003 | 1.258 | 2.815 |
| 219510_at | POLQ | 0.042 | 0.036 | 1.587 | 3.132 |
| 218849_s_at | PPP1R13L | 0.004 | 0.001 | 2.513 | 1.148 |
| 230015_at | PRCD | 0.020 | 0.014 | 1.738 | 2.032 |
| 205053_at | PRIM1 | 0.025 | 0.019 | 1.849 | 3.042 |
| 213951_s_at | PSMC3IP | 0.003 | 0.001 | 2.071 | 3.619 |
| 212662_at | PVR | <0.001 | <0.001 | 2.458 | 1.896 |
| 227123_at | RAB3B | <0.001 | <0.001 | 2.090 | 1.402 |
| 239202_at | RAB3B | 0.002 | <0.001 | 2.095 | 1.516 |
| 204146_at | RAD51AP1 | 0.014 | 0.009 | 2.439 | 6.474 |
| 223322_at | RASSF5 | 0.010 | 0.006 | 2.007 | 1.478 |
| 204127_at | RFC3 | 0.026 | 0.020 | 1.626 | 2.660 |
| 204128_s_at | RFC3 | 0.047 | 0.041 | 1.472 | 2.305 |
| 203022_at | RNASEH2A | 0.004 | 0.002 | 1.681 | 2.778 |
| 226028_at | ROBO4 | 0.025 | 0.019 | 1.018 | 2.334 |
| 235309_at | RPS15A | 0.002 | <0.001 | 2.048 | 1.907 |
| 238824_at | RPS29 | 0.001 | <0.001 | 2.021 | 1.730 |
| 201476_s_at | RRM1 | 0.001 | <0.001 | 1.607 | 2.590 |
| 201477_s_at | RRM1 | 0.016 | 0.011 | 1.698 | 2.753 |
| 201890_at | RRM2 | 0.011 | 0.006 | 3.845 | 10.073 |
| 209773_s_at | RRM2 | 0.012 | 0.008 | 3.276 | 8.878 |
| 219684_at | RTP4 | 0.012 | 0.007 | 2.844 | 1.854 |
| 242845_at | RUNX1T1 | 0.017 | 0.012 | 2.194 | 1.570 |
| 228176_at | S1PR3 | 0.003 | <0.001 | 2.409 | 1.284 |
| 230464_at | S1PR5 | 0.015 | 0.010 | 2.193 | 1.224 |
| 219493_at | SHCBP1 | 0.008 | 0.004 | 2.625 | 7.541 |
| 217640_x_at | SKA1 | 0.012 | 0.007 | 1.891 | 5.256 |
| 227165_at | SKA3 | 0.024 | 0.018 | 1.463 | 2.738 |
| 222217_s_at | SLC27A3 | 0.002 | <0.001 | 2.106 | 1.774 |
| 235050_at | SLC2A12 | <0.001 | <0.001 | 3.950 | 1.987 |
| 244353_s_at | SLC2A12 | <0.001 | <0.001 | 4.695 | 2.542 |
| 223044_at | SLC40A1 | <0.001 | <0.001 | 6.691 | 3.683 |
| 235518_at | SLC8A1 | 0.001 | <0.001 | 1.591 | 2.200 |
| 222727_s_at | SLC8B1 | 0.036 | 0.030 | 2.010 | 1.329 |
| 213624_at | SMPDL3A | <0.001 | <0.001 | 2.007 | 1.488 |
| 209427_at | SMTN | 0.003 | <0.001 | 2.305 | 1.857 |
| 239222_at | SPACA9 | 0.035 | 0.029 | 1.579 | 2.287 |
| 203145_at | SPAG5 | 0.011 | 0.007 | 2.102 | 3.552 |
| 212558_at | SPRY1 | <0.001 | <0.001 | 3.323 | 1.702 |
| 230212_at | SPRY1 | 0.002 | <0.001 | 2.714 | 1.852 |
| 204595_s_at | STC1 | 0.019 | 0.014 | 2.326 | 5.279 |
| 235483_at | STX3 | 0.005 | 0.002 | 1.847 | 2.541 |
| 202565_s_at | SVIL | 0.009 | 0.005 | 2.278 | 1.278 |
| 223274_at | TCF19 | 0.003 | 0.001 | 2.014 | 3.535 |
| 231867_at | TENM2 | 0.008 | 0.004 | 2.784 | 2.248 |
| 206117_at | TPM1 | <0.001 | <0.001 | 2.812 | 1.263 |
| 204985_s_at | TRAPPC6A | 0.001 | <0.001 | 2.090 | 2.067 |
| 205150_s_at | TRIL | 0.002 | <0.001 | 7.960 | 2.438 |
| 204033_at | TRIP13 | 0.003 | <0.001 | 2.125 | 4.246 |
| 217979_at | TSPAN13 | 0.001 | <0.001 | 4.325 | 1.953 |
| 214606_at | TSPAN2 | 0.003 | 0.001 | 4.807 | 1.010 |
| 209372_x_at | TUBB2B///TUBB2A | 0.013 | 0.008 | 1.747 | 2.137 |
| 209191_at | TUBB6 | <0.001 | <0.001 | 2.216 | 1.768 |
| 201714_at | TUBG1 | <0.001 | <0.001 | 1.872 | 2.232 |
| 202589_at | TYMS | 0.018 | 0.013 | 1.324 | 2.799 |
| 223229_at | UBE2T | 0.002 | <0.001 | 2.429 | 3.221 |
| 225655_at | UHRF1 | 0.021 | 0.015 | 2.838 | 3.507 |
| 219740_at | VASH2 | 0.002 | <0.001 | 8.295 | 2.542 |
| 216228_s_at | WDHD1 | 0.003 | <0.001 | 1.680 | 2.754 |
| 212423_at | ZCCHC24 | 0.001 | <0.001 | 2.057 | 1.319 |
| 214761_at | ZNF423 | 0.013 | 0.008 | 2.362 | 2.100 |
| 204026_s_at | ZWINT | 0.004 | 0.002 | 2.224 | 4.237 |
| 1559455_at |  | <0.001 | <0.001 | 2.600 | 2.548 |
| 231097_at |  | <0.001 | <0.001 | 2.187 | 2.435 |
| 235613_at |  | 0.003 | <0.001 | 2.127 | 1.673 |
| 235655_at |  | 0.005 | 0.002 | 2.240 | 1.403 |
| 239262_at |  | <0.001 | <0.001 | 4.067 | 2.508 |
| 239723_at |  | 0.008 | 0.004 | 11.101 | 5.355 |
| 240879_x_at |  | 0.012 | 0.007 | 3.632 | 3.609 |

CCCM, cancer-cell-conditioned medium; Corr, Pearson’s correlation coefficient.
