# Supplementary figures and images for: Novel stromal biomarker screening in pancreatic cancer patients using the in vitro cancer-stromal interaction model
Source: BMC Gastroenterol. 2020 Dec 9;20:411. doi: 10.1186/s12876-020-01556-w (PMC7724826; doi:10.1186/s12876-020-01556-w)

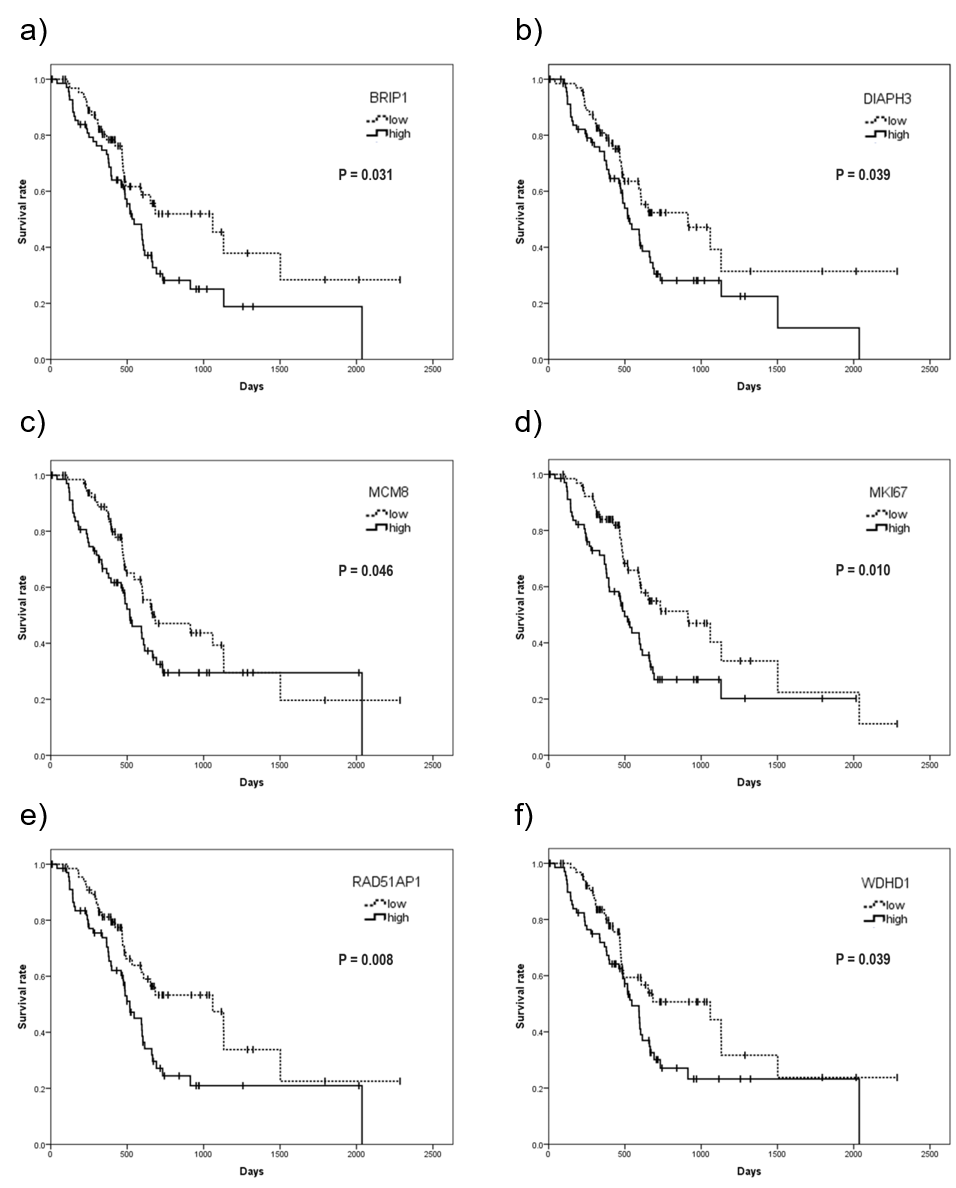

Supplement: Supplementary file 2 — Additional file 2: Figure 1. Kaplan-Meier and log-rank analysis using the TCGA RNA-Seq dataset. After survival analysis of 188 probes using the TCGA data, six genes were selected as prognostic makers: (a) BRIP1 (p = 0.031), (b) DIAPH3 (p = 0.039), (c) MCM8 (p = 0.046), (d) MKI67 (p = 0.01), (e) RAD51AP1 (p = 0.008) and (f) WDHD1 (p = 0.039). TCGA, The Cancer Genome Atlas; RNA-seq, RNA sequencing. [file 12876_2020_1556_MOESM2_ESM.tif]
