# Supplementary material for: Novel stromal biomarker screening in pancreatic cancer patients using the in vitro cancer-stromal interaction model
Source: BMC Gastroenterol. 2020 Dec 9;20:411. doi: 10.1186/s12876-020-01556-w (PMC7724826; doi:10.1186/s12876-020-01556-w)
Supplement: Supplementary file 3 — Additional file 3: Table 2. The following primers were used for the qRT-PCR method [file 12876_2020_1556_MOESM3_ESM.docx]

Supplementary Table 2

The following primers were used for the qRT-PCR method

| Gene Symbol |  | Forward |  | Reverse |
| --- | --- | --- | --- | --- |
| BRIP1 |  | CAAGCATACAGGGCCTTAAACCA |  | TCTGCTGCCGTACCCATTTAGAA |
| DIAPH3 |  | TGAACCTGAGCAGTTTGTGGTTG |  | CCATGATGTCAGGTTTGATGTTG |
| MCM8 |  | CGAGCAAGGTTGGAATTGAGA |  | AGCAACGTTGTTGAGAGCAGA |
| WDHD1 |  | CTCGCTTCACTACAAATGCAAACC |  | GCTGCTATCCATCACATCCACAA |
| GAPDH |  | GCACCGTCAAGGCTGAGAAC |  | ATGGTGGTGAAGACGCCAGT |

qRT-PCR, Real-Time Quantitative Reverse Transcription PCR.
