# Supplementary material for: Novel stromal biomarker screening in pancreatic cancer patients using the in vitro cancer-stromal interaction model
Source: BMC Gastroenterol. 2020 Dec 9;20:411. doi: 10.1186/s12876-020-01556-w (PMC7724826; doi:10.1186/s12876-020-01556-w)
Supplement: Supplementary file 4 — Additional file 4: Table 3. Association of clinicopathological parameters and stromal DIAPH3 expression of 216 PDAC patients in our cohort [file 12876_2020_1556_MOESM4_ESM.docx]

Supplementary Table 3

Association of clinicopathological parameters and stromal DIAPH3 expression of 216 PDAC patients in our cohort

|  | Stromal DIAPH3 strong  n=131 | Stromal DIAPH3 weak  n=85 | P value |
| --- | --- | --- | --- |
| Gender  male/female | 86/45 | 53/32 | 0.621 |
| Age, years* | 67.0 (34–83) | 67.3 (37–84) | 0.947 |
| Tumor size, mm* | 30.0 (10.0–78.0) | 28.0 (11.0–55.0) | 0.217 |
| Location, head of pancreas | 84 | 60 | 0.325 |
| Histological differentiation |  |  | 0.167^a^ |
| Well | 23 | 22 |  |
| Mod | 103 | 59 |  |
| Por | 5 | 4 |  |
| Lymph node metastasis | 100 | 54 | 0.042 |
| Lymphatic invasion, moderate-severe | 30 | 19 | 0.925 |
| Vascular invasion, moderate-severe | 67 | 46 | 0.668 |
| Neural invasion, moderate-severe | 115 | 81 | 0.063 |
| TNM stage |  |  |  |
| 1A | 1 | 1 |  |
| 1B | 1 | 0 |  |
| 2A | 25 | 29 |  |
| 2B | 96 | 54 |  |
| 3 | 2 | 0 |  |
| 4 | 5 | 0 |  |
| R1-2 | 11 | 8 | 0.797 |
| Adjuvant chemotherapy | 80 | 62 | 0.072 |
| Preoperative CA19-9, U/mL* | 105.6 (0.1–53820.0) | 80.4 (0.1–22100.0) | 0.349 |
| Preoperative CEA, ng/mL* | 3.2 (0.7–43.7) | 2.8 (0.7–282.8) | 0.240 |

*median (range)

^a^ Well vs. Mod/Por

DIAPH3, Diaphanous-related formin-3; PDAC, pancreatic ductal adenocarcinoma.
