# Supplementary material for: Novel stromal biomarker screening in pancreatic cancer patients using the in vitro cancer-stromal interaction model
Source: BMC Gastroenterol. 2020 Dec 9;20:411. doi: 10.1186/s12876-020-01556-w (PMC7724826; doi:10.1186/s12876-020-01556-w)
Supplement: Supplementary file 5 — Additional file 5: Table 4. Selected probe sets by gene expression analysis of fibroblasts (NFs and PFs) with pancreatic CCCM stimulation [file 12876_2020_1556_MOESM5_ESM.docx]

Supplementary Table 4

Selected probe sets by gene expression analysis of fibroblasts (NFs and PFs) with pancreatic CCCM stimulation

| NFs | | PFs | |
| --- | --- | --- | --- |
| Probe Set ID | Gene Symbol | Probe Set ID | Gene Symbol |
| 204348_s_at | AK4///LOC100507855 | 215728_s_at | ACOT7 |
| 225342_at | AK4///LOC100507855 | 202274_at | ACTG2 |
| 230630_at | AK4///LOC100507855 | 229585_at | ADAMTSL1 |
| 221009_s_at | ANGPTL4 | 237217_at | ADAMTSL1 |
| 242727_at | ARL5B | 239909_at | ADAMTSL1 |
| 201848_s_at | BNIP3 | 1559982_s_at | AKR1E2 |
| 201849_at | BNIP3 | 207522_s_at | ATP2A3 |
| 226313_at | C10orf35 | 206866_at | CDH4 |
| 242555_at | C16orf87 | 220227_at | CDH4 |
| 235979_at | C7 | 205533_s_at | CDH6 |
| 208650_s_at | CD24 | 222848_at | CENPK |
| 209771_x_at | CD24 | 228559_at | CENPN |
| 205532_s_at | CDH6 | 226610_at | CENPV |
| 222848_at | CENPK | 229610_at | CKAP2L |
| 235117_at | CHAC2 | 210689_at | CLDN14 |
| 229610_at | CKAP2L | 203951_at | CNN1 |
| 221698_s_at | CLEC7A | 227209_at | CNTN1 |
| 218498_s_at | ERO1L | 235706_at | CPM |
| 202345_s_at | FABP5 | 206315_at | CRLF1 |
| 244650_at | FAM105A | 229097_at | DIAPH3 |
| 1555136_at | FGD6 | 1555136_at | FGD6 |
| 200959_at | FUS | 205117_at | FGF1 |
| 207014_at | GABRA2 | 208240_s_at | FGF1 |
| 214240_at | GAL | 1552721_a_at | FGF1 |
| 203178_at | GATM | 240042_at | FIBCD1 |
| 227695_at | GLYATL1///LOC100287413 | 1570515_a_at | FILIP1 |
| 206432_at | HAS2 | 203359_s_at | GJA9-MYCBP///MYCBP |
| 230372_at | HAS2 | 204221_x_at | GLIPR1 |
| 203821_at | HBEGF | 204222_s_at | GLIPR1 |
| 216302_at | HNRNPCL1///LOC440563///LOC649330 | 214085_x_at | GLIPR1 |
| 203828_s_at | IL32 | 226136_at | GLIPR1 |
| 206969_at | KRT34///LOC100653049 | 226142_at | GLIPR1 |
| 1555673_at | KRTAP2-4///LOC730755 | 206432_at | HAS2 |
| 202267_at | LAMC2 | 244764_at | HIVEP3 |
| 205266_at | LIF | 223689_at | IGF2BP1 |
| 1556834_at | LOC100652770 | 203828_s_at | IL32 |
| 200737_at | LOC100652805///LOC100653302///PGK1 | 233533_at | KRTAP1-5 |
| 227068_at | LOC100652805///LOC100653302///PGK1 | 211456_x_at | MT1P2 |
| 226346_at | MEX3A | 211091_s_at | NF2 |
| 230710_at | MIR210HG | 218915_at | NF2 |
| 236480_at | MIR210HG | 215463_at | OR7E24 |
| 204745_x_at | MT1G | 206825_at | OXTR |
| 224399_at | PDCD1LG2 | 224399_at | PDCD1LG2 |
| 218644_at | PLEK2 | 214866_at | PLAUR |
| 211771_s_at | POU2F2 | 212662_at | PVR |
| 208511_at | PTTG3P | 228153_at | RNF144B |
| 1558021_at | RABEPK | 206421_s_at | SERPINB7 |
| 201249_at | SLC2A1 | 243681_at | SHANK2 |
| 240419_at | SLC6A15 | 219090_at | SLC24A3 |
| 204467_s_at | SNCA | 1558920_at | SLC8A1-AS1 |
| 219888_at | SPAG4 | 225728_at | SORBS2 |
| 206434_at | SPOCK3 | 217979_at | TSPAN13 |
| 220030_at | STYK1 | 201714_at | TUBG1 |
| 217704_x_at | SUZ12P | 223229_at | UBE2T |
| 201645_at | TNC | 238542_at | ULBP2 |
| 223229_at | UBE2T | 208081_s_at | ZNF442 |
| 240086_at | VPS36 |  |  |

NFs, normal fibroblasts; PFs, pancreatic fibroblasts; CCCM, cancer-cell-conditioned medium.
